# Transactions of the American Medical Association

**Published:** 1856-01

**Authors:** 


*Transactions of the American Medical Association.*—We are pleased to note that this document is yearly increasing in interest and value. In hastily running over its pages, we found the original articles to be of the first importance, because of the subjects discussed, and by those too from whom we may naturally look for substantial matter.

This volume came to us at a time when our close engagements would forbid a careful analysis, nor have we been able since to peruse it with that care it deserves.

The publishing committee deserve much credit for the manner in which they have discharged their duty, in making this volume superior to any of its predecessors, in point of execution.

Several interesting reports were excluded from this volume, because inconsistent with the established rules of the Association. Dr. Fenners' came under this rule, which we much regret.

